# Recurrent left ischemic stroke in a patient with systemic lupus erythematosus: A case report

**DOI:** 10.1002/ccr3.8192

**Published:** 2023-11-15

**Authors:** Ananda Aryal, Anita Aryal, Dipika Kshetri, Sagun Karki, Sabina Khadka

**Affiliations:** ^1^ All Nepal Hospital Kathmandu Nepal; ^2^ Department of Physiotherapy Kathmandu University School of Medical Sciences Dhulikhel Nepal; ^3^ College of Medicine Nepalese Army Institute of Health Sciences Kathmandu Nepal

**Keywords:** ischemia, MCA, physiotherapy, SLE, stroke

## Abstract

**Key Clinical Message:**

Some studies have manifested the relationship between SLE and stroke. Therefore, it is very important to pay close attention to the diagnosis of SLE in recurring stroke. In our case, her recurrent stroke attack might be due to the undiagnosed cause of SLE.

**Abstract:**

An ischemic stroke is a medical emergency condition which occurs when blood supply to part of the brain is blocked. Early action is needed; therefore, time is crucial. SLE is an autoimmune disease with multiple joint pain, fever, rashes, and organ damage. We report an old lady who was recently diagnosed with SLE with multiple stroke attack. Although she was diagnosed with SLE much later there was a suspicious about the possible mechanism for her recurrent ischemic stroke. She was given antiplatelet, antiepileptic, antihypertensive, and hydroxychloroquine medicines for the treatment. The neurological symptoms improved only after we provided physiotherapy.

## 1 INTRODUCTION

World Health Organization defined stroke as “rapidly developed clinical signs of focal or global disturbance of cerebral function, lasting more than 24 h or leading to death, with no apparent cause other than of vascular origin”. Stroke is one of the major causes of disability and is considered the second leading cause of death worldwide. Stroke mortality grew to 43% from 1990 to 2019, while the incidence of stroke increased by 70% over that time.^1^ In contrast, the death rate in Nepal comprises 42% of all deaths and is expected to rise to 66.3% by 2030.^2^, ^3^, ^4^ Following that the worldwide factsheet released in 2022 confirmed that over the past 17 years, the prevalence of stroke has increased by 50% and it is currently projected that one in four persons experience a stroke during their lifetime and women have a higher lifetime risk of stroke than men (1 in 5 vs. 1 in 6).^5^ Among them, the global burden of disease (GBD) estimates that over 7.6 million new ischemic strokes are accounted for annually, and over 62% of all incident strokes are ischemic strokes worldwide.^1^ Despite having different advanced management strategies for stroke and the use of secondary prevention, recurrent stroke still becomes a challenging illness to address.^6^ According to studies, recurrence rates ranges from 7% to 20% after 1 year to 16% to 35% in 5 years^7^, ^8^ which is still high in number.

Stroke can be caused by different medical and personal factors including high blood pressure, diabetes mellitus, high cholesterol level and lipids, smoking habits, lack of physical activity, dietary habits, and family history. Studies also show that patients with systemic lupus erythematosus (SLE), which is a multi‐systemic autoimmune disease, have a higher chance of having a stroke above 50 years (up to 10‐fold).^9^ The incidence ranges from 5.8 to 25.3 new cases per 1000 person years.^9^ Therefore individuals with SLE are at greater risk of developing stroke than the general population, and the condition needs to be addressed. Here we present a 70‐year‐old age female with SLE with a recurrent large vessel ischemic stroke and review the literature to identify the demographic, clinical, imaging, and outcome characteristics of such presentations.

## 2 CASE REPORT

### 2.1 Patient Information

A 70‐year‐old woman, with a known case of hypertension and ischemic stroke presented to the hospital with a complaint of right‐sided weakness. The patient was found slumped over the bed at home on December 23, 2022 by her son around noon. Soon after weakness, she had one episode of vomiting and abnormal lower body movement. Her son managed to take her to the hospital 7 h later where she was managed symptomatically.

### 2.2 Clinical findings

At the time of admission, her GCS score was 12, and oxygen saturation was maintained at 95% at 1 L per minute of oxygen supply. Throughout the hospital stay her blood pressure remains 150/90 mm of hg and HR at 62 bpm. On chest auscultation, she demonstrated normal vesicular breath sounds and had no abnormality. On observations, she had no muscle wasting and had external rotation deformities seen on the right lower limb. Other neurological evaluations revealed Broca's aphasia on cranial nerve examination. Muscle power examination revealed normal strength on the left side but weakness in the right upper and lower limb. She had a mute right planter reflex and left upgoing plantar reflex.

### 2.3 Past medical history

She had a history of hypertension for 9 years and is under antihypertensive medicine. She experienced three previous episodes of left ischemic stroke in 2014, 2019, and 2021. She was diagnosed with SLE in 2022.

On the first attack, the patient had a sudden loss of consciousness for about 2 h associated with facial deviation to the left side on July 7, 2014. The patient's party had taken her to the hospital which took around 13 h. Computed tomography (CT) was done at the hospital and diagnosed with left ischemic middle cerebral artery (MCA) stroke. The patient had right‐sided weakness and aphasia. However, she had good motor and speech recovery with physiotherapy and speech therapy, respectively.

The patient on of March 1, 2019 again had second episodes of sudden weakness in the right side of the body and slurring of speech. Patient attendants took her to the hospital which took about 2 h and again took her to the hospital where CT was done and revealed that acute on chronic left MCA territory infarct. The patient was managed conservatively at the hospital and received anticoagulants, statins, and antihypertensive medications. She was discharged from the hospital with oral medications, and she had a good recovery in a week.

Again, on December 22, 2021, she had similar weakness in the right half of her body for about a week. CT was done which showed ischemic stroke with involvement of MCA artery territories. She recovered after a week and get discharged.

## 3 DIAGNOSTIC ASSESSMENT

**FIGURE 1:**
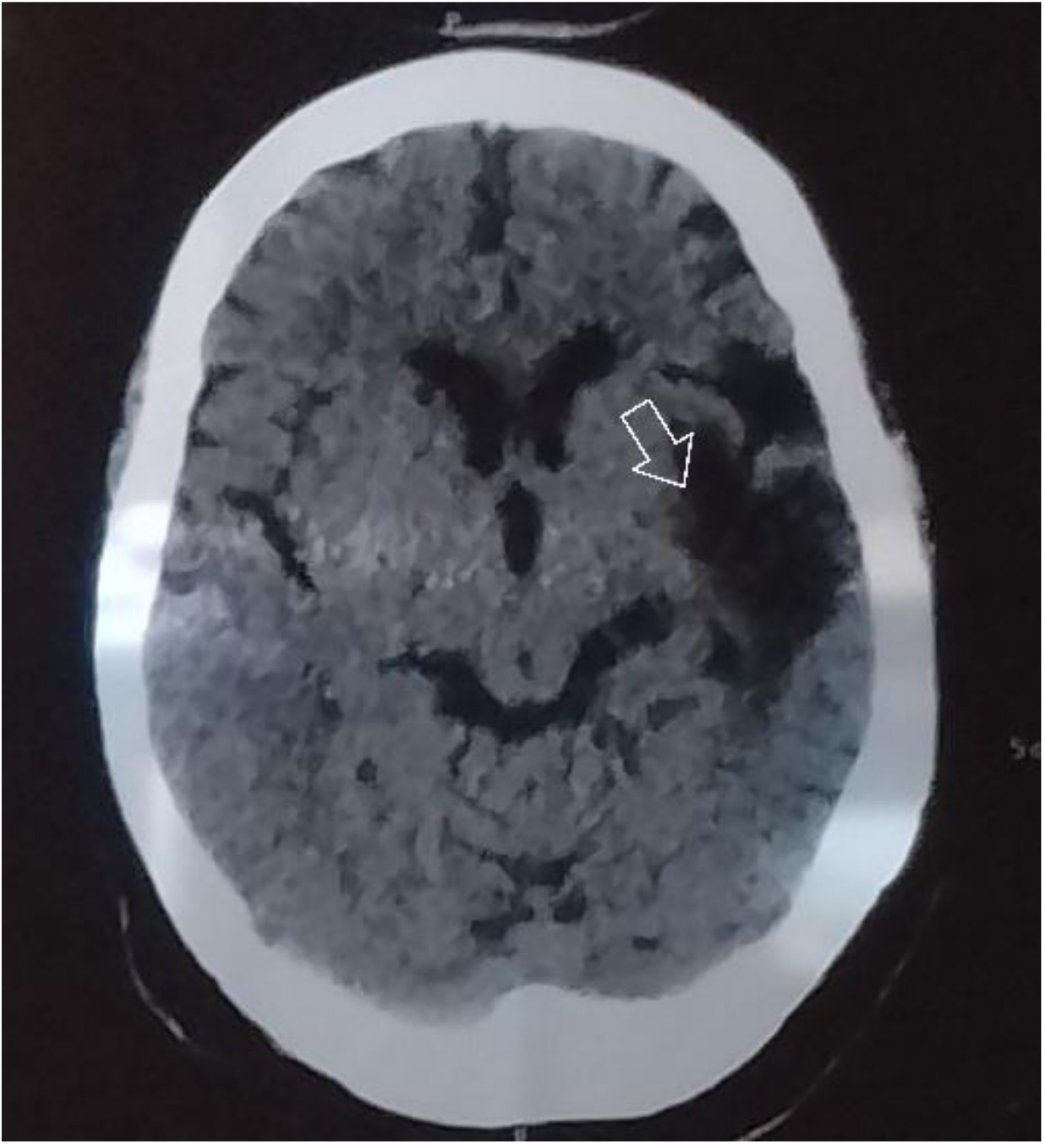
Head CT showing areas of infarction as described in the text.

CT scan done on February 1, 2023 revealed chronic left anterior cerebral artery (ACA) territory infarction involving the left medial frontal lobe along with chronic left MCA territory infarction involving the left frontal and left parietal lobes in the region of the superior division of left M2 MCA focal encephalomalacia with gliosis left MCA territory involving the left frontal temporal and parietal lobes likely due to old infarction as shown in Figure 1.

All baseline investigations were also sent which were found to be normal. However, she was tested for ANA and dsDNA positive. The laboratory results are reported in Table 1.

**TABLE 1:** Clinical biochemical and hematology blood results of the patient.

Hb	13.1 gm%
WCC	5320 cell/cumm
Platelet count	224 000 cell/cumm
MCV	28.9
Neutrophils	68%
Lymphocytes	30%
INR	1
PT	10
APTT	22
Sodium	138 mg/dL
Potassium	3.7 mEq/L
Urea	24 mg/dL
Creatinine	0.5 mg/dL
Serum Random glucose	128 mg/dL
T.Bilirubin	0.8 mg/dL
Albumin	4.36 g/dL
Total protein	7.28 g/dL
ANA	Positive
dsDNA	Positive

Abbreviations: ANA, Antinuclear antibody; APTT, Activated partial thromboplastin time; CRP, C‐reactive protein; Hb, Hemoglobin; INR, International normalized ratio; MCV, Mean corpuscular volume; PT, Prothrombin time; WCC, White cell count.

## 4 THERAPEUTIC INTERVENTION

### 4.1 Medical treatment

On the current admissions, this patient was managed with oxygen at 1 L per min via nasal cannula. Her vitals were stable, and she was managed accordingly with good recovery during her hospital stay. We prescribed combination of immunosuppressant and antiplatelet therapy, because of the high activity of SLE and the high frequency of stroke recurrence after the initial vascular event. The patient was treated with antiplatelet, antiepileptic, antihypertensive, and hydroxychloroquine. She was also advised physiotherapy.

### 4.2 Physiotherapy

The patient was treated with the passive range of motion (PROM) exercises at first, assisted bridging exercises, rolling and bed mobility exercises, and supported sit‐to‐stand exercises were done. At first, the patient was unable to do all the exercises on the affected side so the intervention focus on the passive along with assisted exercises wherever it seems possible. The treatment session was once a day which lasts for about 1 h in a daily schedule for about 3 months. The patient showed much improvement as she was able to do her bed mobility independently, able to strengthen the muscles of thighs and core with the support of parallel bars, and able to stand without support. Then the treatment session changed to twice a day and more challenging tasks were added to her treatment protocol including squatting without support, gait training with the support of a stick, and so on. Now the patient could walk indoors and outdoors with the support of a stick but need supervision as she loses balance on uneven surfaces.

## 5 OUTCOME AND FOLLOW UP

The patient had no movement in the right upper and lower limbs. She had good static sitting balance but poor dynamic sitting balance. She was able stand with the support of one person along with the support of a hand bar. She could not walk independently. On the objective measurement, we took the Berg Balance scale, which measures the quality of balance in patients while sitting and standing at the beginning and after treatment. At the time of assessment, her balance score was 8 out of 56 which means she had a mobility by wheelchair. However, after physiotherapy her score rose to 23 where she could walk through assistance. Furthermore, we had done Timed Up and Go test which is a simple and widely used clinical test for the assessment of lower extremity function, balance, mobility, and fall risk after 5 months of physiotherapy where she scored 58 s indicating she had a risk of fall. (Table 2).

**TABLE 2:** Outcome of physiotherapy.

Outcome measures	Assessment	After physiotherapy treatment
Berg balance score	8/56	23/56
Timed up and go test	She could not be able to perform at the time of assessment.	58 s

### 5.1 Timeline

**FIGURE 2:**
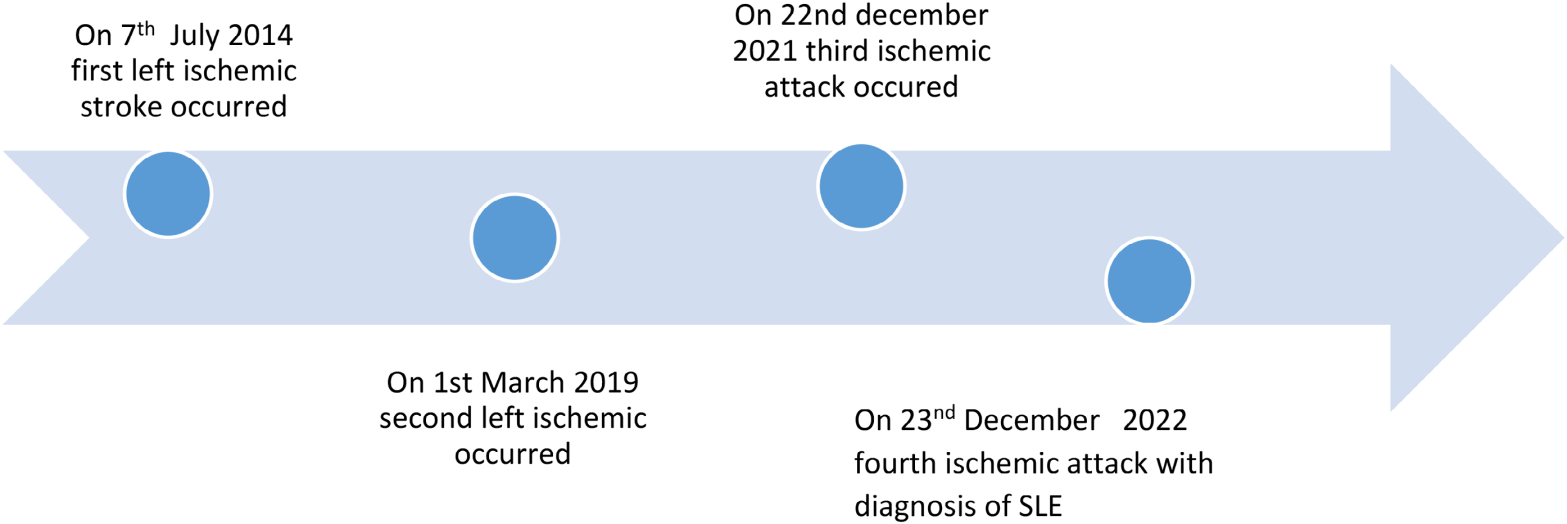
Timeline of events occurring over a period of 9 years.

Clinical evolution of the patient span in the duration of 9 years. The major clinical events that happened over these 10 years are summarized in the timeline. Figure 2.

## 6 DISCUSSION

In this report, we present a 70‐year‐old female who had a recurrent stroke and diagnosed with SLE. The overall rate of recurrent stroke in SLE patients is 3%–20%.^10^ She had four episodes of stroke since 2014 and recently in December 2022. She was later diagnosed with SLE. However, she has joint pain and myalgia for 4 years. Therefore, she could be an undiagnosed case of SLE. SLE is a systemic autoimmune disease with a broad range of clinical manifestations with several phenotypes, ranging from mild mucocutaneous to multiorgan and severe central nervous system involvement.^11^ The causes are unknown; however, several genetics, immunological, endocrine, and environmental factors are postulated. It had been described that in SLE, ischemic stroke is occurred by several different mechanisms, including cardioembolism, large artery stenosis of either non‐atherosclerotic or atherosclerotic etiology, arterial dissection, hypercoagulable states like APLA syndrome and rarely cerebral vasculitis.^12^ Several studies have shown that the risk of ischemic and hemorrhagic stroke is markedly higher in SLE when compared to the general population.^11^ In SLE, there is the deposition of immune complexes within the vessel wall and the formation of antibodies against endothelial cells.^13^ This autoantibody thus can induce endothelial activation which is responsible for the vasculitis^14^ and thus increases the risk of stroke. The highest relative risk of stroke was observed at younger ages, which may be partially explained by the accelerated and premature atherosclerosis seen in SLE.^9^

In our case study, the patient had recurrent ischemic cerebrovascular attacks within 9 years. These reoccurrence attacks were probably related to SLE and other classic risk factors like hypertension, dyslipidemia, and a sedentary lifestyle. Though the patient had recurrent multiple attacks of stroke the diagnosis of SLE was made much later. This helps to provide insight to the physicians about the early possible intervention and diagnosis. She was ANA, anti‐dsDNA antibody positive, and also had multiple joint involvement most commonly shoulder, wrist, knee, and also myalgia. Joint involvement is the most common manifestation of SLE and has been noted around 90% of the patient.^15^ The diagnosis of SLE in our patient fulfilled both clinical and immunological criteria of new ACR and EULAR.^15^

Recurrent stroke encountered with multiple problems of the patient makes the treatment progression more challenging, but the patient seems cooperative and involved in physiotherapy sessions actively. Despite being a more challenging case, physiotherapy treatment along with medical management plays a significant role in achieving the functional level of patients. Intensive physiotherapy intervention includes a routine exercise program to enhanced the functional status and promote neuroplasticity.^16^ In addition to this, the use of antiplatelet and antihypertensive drugs equally promoted to reduce the occurrence of stroke in patients. The AHA/ASA guidelines recommend the use of antiplatelet agents to reduce the risk of a recurrence event.^17^ Studies also suggest that the reduction rate of recurrent ischemic stroke is approximately 22% with the use of antiplatelet therapy.^18^

## 7 CONCLUSION

Early intervention and diagnosis of patients with mild SLE could be challenging. However patients with recurrent stroke correlating with sex, and symptoms should always raise suspicion for underlying SLE, and prompt diagnostic investigation to confirm or exclude its presence helps assist in the progression of the patient's condition and reduce future risk. Essentially, stroke may even be the presenting manifestation of lupus, and patients with SLE may suffer a stroke at even a young age.^9^ Furthermore, intensive neuro‐rehabilitation plays a vital role in the management of patients with recurrent stroke.^18^

## AUTHOR CONTRIBUTIONS

**Ananda Aryal:** Conceptualization; methodology; resources; writing – review and editing. **Anita Aryal:** Formal analysis; software; writing – review and editing. **Dipika Kshetri:** Data curation; investigation. **Sagun Karki:** Project administration; supervision; writing – original draft. **Sabina Khadka:** Conceptualization; data curation; writing – review and editing.

## CONFLICT OF INTEREST STATEMENT

The authors declare no conflict of interest.

## FUNDING INFORMATION

None.

## CONSENT

Written informed consent was obtained from the patient to publish this report in accordance with the journal's patient consent policy.

## Data Availability

Data available on request from the auther.
